# Olfactory models as a platform for neuroscience research

**DOI:** 10.3389/fncel.2026.1897347

**Published:** 2026-08-20

**Authors:** María Figueres-Oñate

**Affiliations:** Instituto de Investigación Enfermedades Raras (IIER), Instituto de Salud Carlos III (ISCIII), Madrid, Spain

**Keywords:** nasal swab sampling, neural progenitors, neurogenesis, olfactory epithelium, olfactory organoids, patient derived models, precision medicine

## Abstract

The capacity of the olfactory epithelium (OE) to sustain adult neurogenesis offers an unprecedented opportunity to investigate the mechanisms of neural lineage specification, cellular turnover, and tissue regeneration in a human context. Unlike conventional brain organoids, which predominantly model embryonic or fetal neurodevelopment, olfactory organoid systems uniquely provide experimental access to active adult human neurogenic and regenerative dynamics. As the only accessible adult neural stem cell niche that can be repeatedly sampled from living patients, this neuroepithelium opens new paths for generating patient-specific, non-reprogrammed neural models. This review provides an overview of OE-derived *in vitro* systems, detailing their evolution from early neurospheres to advanced three-dimensional (3D) organoids, highlighting their capacity to preserve donor genetic, epigenetic, transcriptomic and proteomic signatures, establishing them as a powerful platform for personalized medicine, disease modeling, and “nose-to-brain” therapeutic discovery.

## Introduction

The olfactory system represents a unique and powerful model for studying fundamental neurobiological processes. As the most evolutionarily ancient sensory system, it displays distinct mechanisms of information processing, with olfactory inputs bypassing the thalamus and directly influencing behavioral and physiological responses (Figueres-Oñate et al., 2014). This anatomical and functional organization, together with its remarkable regenerative capacity, has positioned the olfactory pathway as an attractive platform for investigating neural development, degeneration, and repair.

**Figure 1:**
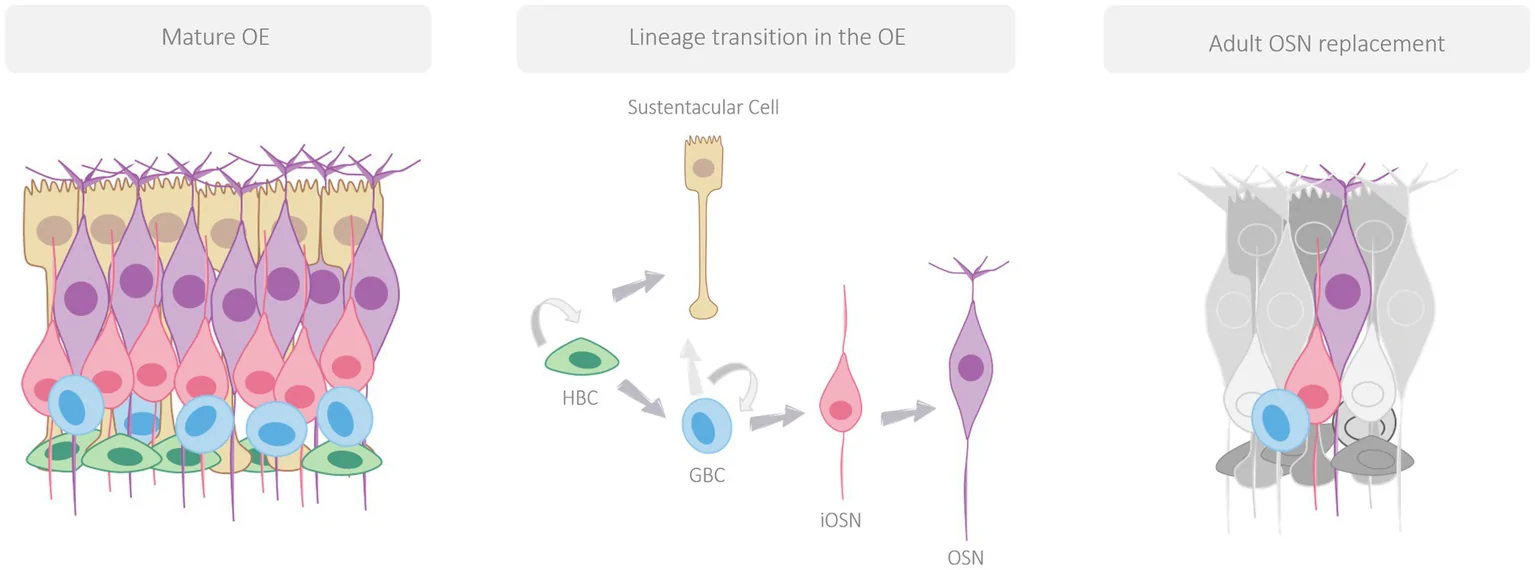
Mature olfactory epithelium (OE, left): schematic representation of the pseudostratified adult olfactory epithelium under homeostatic conditions. The native tissue architecture comprises highly structured apical sustentacular cells (yellow), which provide metabolic and mechanical support; bipolar mature olfactory sensory neurons (OSN, purple) distributed throughout the intermediate layers of the neuroepithelium; immature olfactory sensory neurons (iOSN, red); and a basal layer composed of proliferating globose basal cells (GBC, blue) and quiescent horizontal basal cells (HBC, green) directly anchored to the basement membrane. Lineage transition in the OE (center): quiescent horizontal basal cells (HBC) serve as a reserve stem cell pool capable of self-renewal (circular arrow) and multilineage differentiation into both non-neuronal sustentacular cells and active progenitors. Active neurogenesis is predominantly sustained by the transit-amplifying globose basal cells (GBC), which proliferate to yield immature olfactory sensory neurons OSNi) before committing to terminally differentiated, mature olfactory sensory neurons (OSN). Adult OSN replacement (right): dynamics of life-long neuronal renewal within the established epithelial niche. Highlighted cellular lineages (colored profiles) illustrate the active migration and integration of newly generated sensory neurons (OSNi) into the existing epithelial matrix, replacing aged or damaged cellular elements to maintain steady-state tissue homeostasis and sensory competence.

The first stage of olfactory processing occurs within the olfactory epithelium (OE), a specialized neuroepithelium located in the nasal cavity. The OE comprises multiple cell populations, including olfactory sensory neurons (OSNs), sustentacular cells, basal stem/progenitor cells, and microvillar cells (Figure 1). Basal cells function as resident stem cells, sustentacular glial cells provide structural and metabolic support, and microvillar cells contribute to epithelial homeostasis and immune defense. Among these, OSNs are particularly remarkable, as they represent the only neurons in the human body directly exposed to the external environment. These neurons detect odorants through the expression of a single olfactory receptor (OR) gene, following a monogenic and monoallelic expression pattern that underlies odor discrimination.

Autoradiographic pulse-labeling studies in rodents demonstrated that OSNs undergo continuous turnover throughout adult life, leading to the long-standing view that OSNs have an average lifespan of approximately 30–40 days (Hinds et al., 1984; Mackay-Sim and Kittel, 1991). However, these estimates were derived primarily from tracking newly generated cells and therefore do not necessarily reflect the survival kinetics of the entire mature neuronal population. More recent genetic lineage-tracing approaches, which enable the labeling of approximately 95% of the mature neuron population, revealed that OSNs commonly persisted for up to 6 months after labeling, with neuronal survival varying according to both the age of the animal and anatomical location within the olfactory epithelium (Gaun et al., 2022). Collectively, these findings indicate that OSN turnover is not governed by a single uniform lifespan but instead reflects a spatially and temporally heterogeneous homeostatic process, in which a substantial fraction of mature, established OSNs can persist for many months under steady-state conditions (Brann and Firestein, 2014).

The core concept of lifelong neuronal renewal and robust cellular replacement within the olfactory epithelium remains firmly supported among species, including humans, where histological and molecular studies have confirmed active neurogenesis in adult tissue (Graziadei and Graziadei, 1979; Holbrook et al., 2011). More recently, single-cell transcriptomic analyses have provided a comprehensive characterization of human OE cellular composition and dynamics, revealing that, while the replacement rate of OSNs in adults may be relatively lower than in rodents, the epithelium retains a robust regenerative response (Durante et al., 2020). Importantly, although adult neurogenesis has also been well documented in murine central nervous system niches such as the subventricular zone (SVZ) and hippocampus, its persistence in the adult human brain remains actively debated. In contrast, continuous neuronal replacement in the OE is broadly supported across species. This lifelong plasticity sharply contrasts with the mainly post-mitotic nature of the central nervous system, highlighting the OE as a uniquely accessible model for studying neural regeneration and degeneration.

Neurogenesis within the OE is sustained by a hierarchical organization of basal progenitor populations located at the epithelial base (Figure 1). Two principal stem cell populations have been described: horizontal basal cells (HBCs) and globose basal cells (GBCs). HBCs are largely quiescent under homeostatic conditions, acting as a reserve stem cell population capable of regenerating the entire epithelial lineage following severe injury (Leung et al., 2007). In contrast, GBCs represent the actively proliferating progenitor pool responsible for the generation of new OSNs. The coordinated interaction between these populations ensures both tissue homeostasis and regenerative capacity, establishing the OE as a well-defined adult stem cell niche.

These biological features motivated the use of the OE as a source of living human neural tissue. Beginning in the late 20th century, advances in biopsy techniques allowed the safe and reliable collection of olfactory mucosa from living individuals. Advances in *in vitro* approaches facilitated the processing of olfactory mucosa biopsies to dissociate and isolate cells, enabling the development of cultures. These systems provided one of the first reproducible human neural models obtained without pluripotent stem cell reprogramming, preserving patient-specific molecular signatures to study neurodevelopmental and neurodegenerative disorders in a physiologically relevant human context. Current efforts in the field are directed toward the development of three-dimensional olfactory culture systems, including organoids and emerging assembloid approaches (Chiaradia and Lancaster, 2026), with the aim of more accurately recapitulating tissue architecture, cellular interactions, and functional dynamics.

Building on these advances, this review aims to provide an overview of OE-derived *in vitro* systems. Particular emphasis is placed on their role in modeling adult neurogenesis, disease mechanisms, and patient-specific cellular phenotypes, as well as their emerging potential to form advanced three-dimensional systems. Ultimately, these approaches support the development of “nose-to-brain” platforms for studying disease progression and therapeutic responses.

## Minimally invasive sampling approaches

One of the most distinctive advantages of olfactory epithelium–derived *in vitro* systems is the possibility of obtaining living neural tissue through minimally invasive procedures. Unlike most regions of the human nervous system, the olfactory neuroepithelium is directly accessible from the nasal cavity, enabling repeated sampling without the need for invasive neurosurgical intervention (Mackay-Sim, 2013). This accessibility represents a critical advantage over other human neural models and has significantly expanded the translational potential of olfactory-based systems.

Initial studies relied primarily on surgical biopsies obtained from anatomically defined olfactory regions such as the superior turbinate or posterior nasal septum (Féron et al., 1998; Jafek et al., 2002). Standardization of biopsy and primary culture protocols has been a major determinant of downstream reproducibility. Early methodological frameworks established reproducible procedures for primary culture from adult human OE, transforming it into a reliable source of patient-derived neural material (Rawson and Ozdener, 2013). Endoscopic approaches further increased tissue accessibility for clinical research and were validated as a rich source of neural progenitors, providing the methodological basis for continuously expandable adult human OE-derived cultures (Winstead et al., 2005). More recently, defined culture conditions for human olfactory stem cell expansion, complemented by single-cell transcriptomic validation confirming the olfactory rather than respiratory identity of the resulting cultures, have been reported (Oliva et al., 2022). Quantitative assessments indicate that approximately 70–80% of OE biopsy tissues obtained from the nasal septum or middle turbinate yield olfactory receptor neurons upon immediate dissociation, providing a practical baseline against which protocol efficiency can be calibrated across laboratories (Borgmann-Winter et al., 2015).

While these approaches provided high-quality olfactory tissue enriched in neuroepithelial cells, they required specialized clinical procedures and were associated with limitations including patient discomfort, procedural risk, and restricted feasibility for longitudinal sampling. To overcome these constraints, less invasive approaches were subsequently developed based on nasal brushing and exfoliative sampling techniques (Benítez-King et al., 2011). The development of swab-based and exfoliation protocols simplifies patient sampling and increases accessibility in both clinical and research settings. Although these methods may yield heterogeneous cell populations and variable proportions of olfactory neuroepithelium, optimized protocols have demonstrated successful generation of expandable epithelial cultures and patient-derived models, retaining their capability to generate neurospheres and adherent cultures (Unzueta-Larrinaga et al., 2023).

Minimally invasive nasal sampling approaches have been particularly successful for the generation of respiratory nasal airway organoids, which can be robustly expanded from swab-derived epithelial cells and have rapidly become widely used platforms for studying airway biology, viral infection, inflammation, and personalized therapeutic responses (Chiu et al., 2022; Zhu et al., 2025). In contrast, the establishment of olfactory derived cultures from minimally invasive samples remains considerably more challenging. This limitation primarily reflects the patchy anatomical distribution of the olfactory mucosa in adult humans, the low abundance of olfactory stem/progenitor populations within routine nasal samples, and the frequent co-isolation of respiratory epithelial cells (Wu et al., 2024). Consequently, while nasal airway organoid protocols are becoming increasingly standardized and reproducible, the reliable enrichment and long-term maintenance of true olfactory epithelial cultures still represent a major technical challenge for the field.

In parallel, direct sequencing of nasal swab–derived samples enables the characterization of transcriptomic profiles from olfactory populations, including basal progenitors, sustentacular cells, and immature neuronal populations (Caballero et al., 2025). Notably, patient-derived cultures generated from nasal swabs have been shown to preserve disease-associated transcriptional signatures, reinforcing their potential as a model for personalized medicine (Unzueta-Larrinaga et al., 2026).

Species-specific differences in OE cellular composition have direct consequences for biopsy targeting and culture interpretation. The topological, morphological, and molecular expression patterns of basal cell populations in the human OE are less clearly defined than in the mouse, where HBCs and GBCs are anatomically distinct. Immunophenotypic markers conventionally used to identify these populations in rodent studies therefore cannot be directly transposed to human biopsies, and species-aware validation is necessary when extrapolating culture protocols or molecular signatures among systems. When enzymatic dissociation is not performed, laser-capture microdissection of the neural layer from OE tissue represents a complementary strategy for selective enrichment of neuronal populations from otherwise heterogeneous biopsy material (Lavoie et al., 2017).

The ability to repeatedly collect samples from living individuals provides a unique opportunity to perform longitudinal studies of neural-associated cellular phenotypes. This is particularly relevant for neurodevelopmental and neurodegenerative disorders, where dynamic changes in cellular behavior may occur over time. Moreover, patient-derived olfactory cultures retain aspects of the donor’s epigenetic and molecular identity, allowing investigation of disease-specific phenotypes in a physiologically relevant context without requiring reprogramming.

Despite these advantages, important challenges remain present. Factors such as donor age, inflammation, and sampling depth further contribute to inter-sample heterogeneity. Consequently, standardization of sampling protocols and validation of olfactory identity remain critical for ensuring reproducibility and comparability across studies (Pipolo et al., 2026).

Nevertheless, minimally invasive sampling approaches have transformed the olfactory epithelium into one of the most accessible platforms for studying living human neural cells. They represent a key enabling technology for the development of patient-specific models, bridging basic neuroscience and translational research.

## Primary olfactory cultures

The first *in vitro* approaches aimed at modeling the olfactory neuroepithelium relied on primary cultures derived from human olfactory mucosa biopsies and postmortem tissue (Roisen et al., 2001; Wolozin et al., 1992). These pioneering studies demonstrated that cells isolated from the adult human olfactory mucosa could survive, proliferate, and generate free-floating spheroid structures resembling neurospheres, establishing the conceptual basis for the development of olfactory-derived neural culture systems. Importantly, these observations positioned the olfactory mucosa as one of the few accessible sources of living human neural-like cells.

Early protocols typically involved enzymatic dissociation of olfactory mucosa followed by culture in serum-free media supplemented with epidermal growth factor (EGF) and basic fibroblast growth factor (bFGF/FGF2), conditions previously established for neural stem cell expansion from the central nervous system (Reynolds and Weiss, 1992). In parallel, early rodent OE cultures helped define the surface-marker landscape that later basal cell purification protocols built upon, identifying keratin-positive cell populations and sustentacular subsets within dissociated preparations that provided an important cellular reference framework for subsequent human studies (Pixley, 1992). Successive studies provided evidence that these olfactory-derived cultures contained stem/progenitor-like populations with self-renewal and multilineage differentiation potential (Murrell et al., 2005). Proliferative cell populations formed clonogenic spheroid structures that could be serially expanded *in vitro* (Winstead et al., 2005). Studies in mouse olfactory mucosa further demonstrated that growth factor composition strongly influenced cellular behavior, with bFGF acting primarily as a mitogen for rapidly proliferating progenitors, while EGF promoted survival of stem and precursor populations (Barraud et al., 2007).

Among the technical frameworks that have emerged for modeling the olfactory mucosa, one specific approach has become widely adopted in psychiatric and neurological disease research. In this method, olfactory mucosa biopsies, containing both neuroepithelium and underlying lamina propria, were dissociated and initially propagated in serum-containing media before being transferred to growth factor-supplemented conditions combining EGF and FGF2. Under these conditions, cell clusters attach to the substrate while a subpopulation detaches to form free-floating neurospheres that are multipotent, capable of self-renewal, and can be differentiated into neurons and glia (Lavoie et al., 2017; Mackay-Sim, 2013). This framework has been applied in a wide range of neurological and psychiatric disease contexts and has become one of the most widely reproduced patient-derived neural culture approaches in the field.

A parallel organotypic monolayer tradition has also been described, distinct in its preservation of native epithelial cytoarchitecture. In this approach, non-dissociated OE biopsy tissue, including a portion of underlying lamina propria, is propagated directly in serum-containing media on fibronectin-coated or Matrigel substrates. Epithelial cells migrate out from the biopsy to form sheets, over which a more rapidly proliferating cell population emerges within the first 48 h. This system has been used to interrogate cellular processes relevant to psychiatric disease and supports neuronal differentiation when supplemented with neurotrophins and differentiation-inducing factors (Borgmann-Winter et al., 2015). Compared to dissociated neurosphere protocols, this approach better preserves aspects of the native cellular organization, though it similarly yields heterogeneous populations whose neuroepithelial identity requires validation.

**Figure 2:**
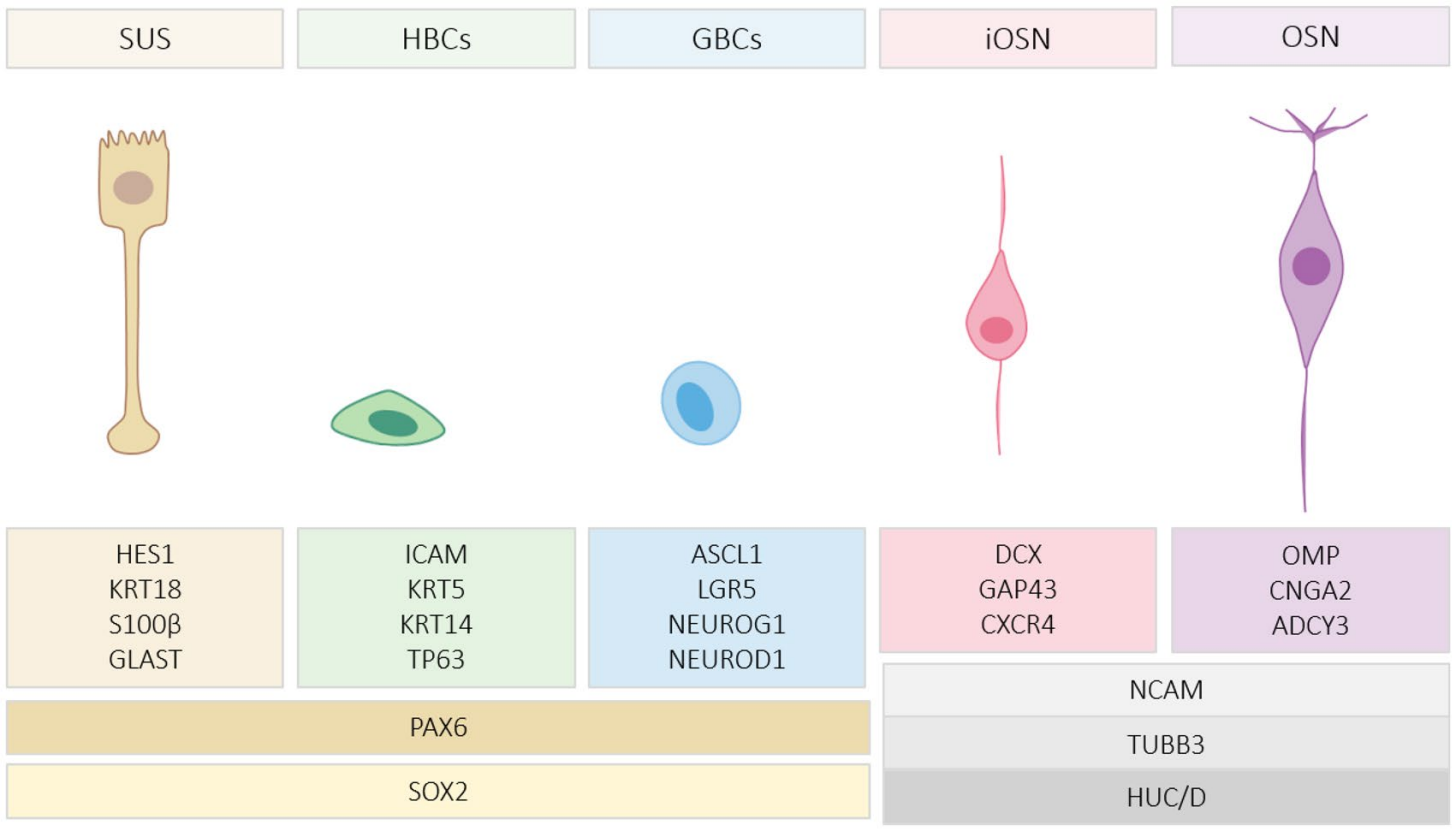
Molecular marker profiles and lineage-specific signatures of olfactory epithelial cell populations. Key cellular stages of the olfactory epithelium (OE) differentiation hierarchy alongside their characteristic transcriptional, structural, and surface markers, serving as a reference panel for validating *in vitro* cultures and organoid models.

The identity of the cells was typically assessed by the classical defined markers for the OE cells lineage (Figure 2). Neurosphere-derived cells expressed markers associated with immature neural identity and stemness, including Nestin, SOX2, Musashi-1, and other progenitor-associated proteins (Zelenova et al., 2021). Upon differentiation, these cultures generated neuron-like and glial-like cells expressing markers such as βIII-tubulin (TUBB3), MAP2, NeuN, GFAP, and S100β (Murrell et al., 2005; Roisen et al., 2001).

Importantly, human olfactory cultures were shown to acquire neuronal characteristics. Biopsy-derived human olfactory epithelial cells expressed functional neurotransmitter receptors, including dopaminergic, serotonergic, and glutamatergic receptors, together with components of olfactory transduction pathways (Borgmann-Winter et al., 2009). These findings provided some of the first evidence that patient-derived olfactory cultures could exhibit functionally relevant neuronal properties beyond simple expression of immature neural markers.

As the field progressed, increasing attention focused on identifying the endogenous stem and progenitor populations responsible for olfactory epithelial regeneration. Studies in rodents established the hierarchical organization of the olfactory stem cell niche, identifying HBCs as largely quiescent reserve stem cells and GBCs as actively proliferating neurogenic progenitors (Leung et al., 2007; Packard et al., 2011; Schwob et al., 2017). Subsequent lineage-tracing and single-cell transcriptomic studies further resolved differentiation trajectories and progenitor heterogeneity within the olfactory epithelium (Fletcher et al., 2017; Ren et al., 2022).

These mechanistic insights enabled the development of more defined culture systems for selective expansion of olfactory basal progenitors. Surface-marker-guided isolation, using ICAM-1-based selection to achieve high-purity HBC enrichment from dissociated OE, demonstrated that individual HBCs could form rapidly expanding colonies under defined matrix and serum conditions, and that the most mitotically active among these displayed multipotency, spontaneously generating presumptive HBCs, olfactory neuroglial progenitors, and neurons (Carter et al., 2004). This work provided the methodological basis for subsequent demonstrations that the reserve HBC population can be activated *in vitro* to support transplantation-engraftable multipotency across mouse, rat, and human systems (Peterson et al., 2019). HBC-enriched cultures maintained TP63^+^/KRT5^+^ basal stem cells under proliferative conditions using extracellular matrix-coated substrates and growth factor-defined media (Fletcher et al., 2017). In parallel, GBC-associated populations isolated through c-KIT or Lgr5-related strategies demonstrated robust neurogenic competence and multilineage differentiation capacity (Chen et al., 2014; Ren et al., 2022). Additional culture systems, including air–liquid interface spheres and colony-based 3D cultures, further improved the maintenance of olfactory epithelial hierarchy and neurogenic potential *ex vivo* (Jang et al., 2008; Ren et al., 2021).

After decades of work, patient-derived olfactory cultures have generated several important conceptual advances in the study of neuropsychiatric and neurological disorders. Olfactory neurosphere-derived cells have been applied to the study of schizophrenia, Parkinson’s disease, autism spectrum disorders, and other neurological conditions using living patient-derived neural tissue. These studies revealed alterations in neurodevelopmental programs, extracellular matrix organization, mitochondrial homeostasis, oxidative stress, and inflammatory pathways, several of which overlap with transcriptomic signatures observed in postmortem brain tissue (Matigian et al., 2010; McCurdy et al., 2006; Nguyen et al., 2016). Patient-derived cultures have shown that inflammatory signaling suppresses progenitor proliferation and neuronal differentiation while redirecting stem-cell programs toward epithelial repair and immune-defense states, providing a plausible mechanism for persistent inflammatory anosmia beyond simple neuronal loss (Liu et al., 2026; Yang et al., 2024). Similarly, aging studies have identified progressive dysfunction of basal stem-cell populations, including altered HBC activation and depletion of GBCs, as a major contributor to the decline in olfactory regenerative capacity (Child et al., 2018; Li et al., 2024). Published studies have largely relied on differential gene expression, protein abundance, or proliferation assays. Establishing direct causal links to disease pathophysiology or identifying therapeutic targets has remained challenging. Findings have also been difficult to replicate across cohorts, in part because of the discussed cellular heterogeneity, since variability in biopsy composition and culture conditions can substantially influence the relative abundance of neuroepithelial and non-neuroepithelial populations. Consequently, these systems are best viewed as patient-specific, perturbation-responsive models that preserve native genetic and epigenetic backgrounds without requiring cellular reprogramming. While they have generated biologically relevant hypotheses and revealed disease-associated cellular states, establishing causal disease mechanisms will require more physiologically representative models capable of functional validation.

Another limitation of primary adherent cell cultures and neurosphere systems is their inability to reproduce the spatial organization and cellular complexity of the native olfactory epithelium. Consequently, differentiation toward mature olfactory sensory neurons remained relatively inefficient in most generated structures. Cultures frequently exhibited heterogeneous cellular identities and progressive loss of neurogenic potential during prolonged expansion. Moreover, although neuron-like phenotypes could be induced, these systems rarely reproduced the pseudostratified architecture, the hierarchical organization of HBC and GBC progenitor populations, niche interactions, or functional maturation characteristic of native olfactory tissue. As a result, they provided limited opportunities to investigate how stem-cell behavior, lineage progression, and cell–cell interactions are coordinated within the intact neurogenic niche. These limitations ultimately motivated the transition toward more advanced three-dimensional olfactory organoid systems capable of better recapitulating tissue architecture, stem cell dynamics, and neuronal differentiation. By preserving multicellular organization and endogenous signaling environments, organoids would offer a more physiologically relevant platform for studying olfactory development, regeneration, disease mechanisms, and patient-specific biology.

## Emergence of olfactory organoids

Initial attempts to generate olfactory organoids were primarily performed using mouse-derived olfactory stem and progenitor populations, including GBCs, HBCs, and Lgr5^+^ cells (Chen et al., 2014; Ren et al., 2022). These models demonstrated the capacity to generate multiple olfactory epithelial cell types, although the degree of structural organization, cellular composition, and reproducibility varied across studies, reflecting the experimental and biological complexity of the system.

More recent work has expanded these approaches in multiple directions, collectively improving aspects of epithelial architecture, lineage representation, and stem cell dynamics (Ren et al., 2021). In particular, dissociated epithelial cells embedded in extracellular matrix scaffolds, such as Matrigel or Cultrex, were shown to self-organize into 3D structures exhibiting some features of pseudostratified epithelium (Gameiro et al., 2025b; Ozgun et al., 2024). Novel derived murine organoid systems have begun to recapitulate aspects of stem cell hierarchy and niche-like interactions within the olfactory epithelium, highlighting distinct functional roles of basal cell populations in regulating neurogenesis (Gameiro et al., 2025a). In these systems, HBC were shown to regulate the niche and GBC were the proliferative ones, partially recapitulating aspects of the native tissue environment (Jang et al., 2008; Schwob et al., 2017). Parallel efforts have focused on optimizing neuronal differentiation and lineage progression in murine models, providing improved characterization of the cellular outputs and culture conditions required for sustained neurogenic potential (Ozgun et al., 2024). In contrast, embryonic progenitor–based approaches generate more organized, pseudostratified epithelial structures that recapitulate features of early morphogenesis and tissue development (Suzuki et al., 2025). These models reproduce key hallmarks of olfactory epithelial organization, including the “one olfactory sensory neuron–one odorant receptor” expression pattern, and give rise to neuronal populations exhibiting subtype-specific molecular profiles.

Taken together, these murine studies illustrate the emergence of multiple, complementary organoid strategies rather than a single standardized model. While all systems demonstrate some degree of self-organization and multi-lineage differentiation, they differ significantly in terms of cell source (adult versus embryonic), biological focus (regeneration versus development), and degree of maturation. This diversity underscores both the versatility of olfactory epithelial cells and the current lack of convergence within the field.

The need for physiologically relevant and patient-specific systems has driven efforts to translate organoid methodologies to human olfactory tissue. Human olfactory organoid-like cultures have been generated from biopsies of regions enriched in olfactory epithelium, such as the superior turbinate (Kim et al., 2025; Luo et al., 2026). Similar to mouse models, dissociated epithelial cells embedded in 3D matrices can form expandable structures expressing olfactory markers and containing a mixture of progenitor and differentiated cell types. However, these human systems are still limited in number, often lack standardization, and exhibit variability in growth efficiency, cellular composition, and differentiation potential.

One of the most significant advances reported in these systems is the partial emergence of functionally relevant olfactory sensory neuron–like cells. Some studies have demonstrated the expression of mature neuronal markers, including olfactory marker protein (OMP), G_olf, and components of the olfactory transduction cascade (Kim et al., 2025). In a limited number of cases, functional responses to odorant stimulation have been detected through intracellular calcium (Ca^2+^) signaling, suggesting a degree of sensory competence. However, these functional properties are typically observed in a subset of cells and remain variable, indicating that full maturation and functional integration are not yet robustly achieved.

Even more limited are olfactory organoid approaches based on human induced pluripotent stem cells (hiPSCs), few studies have focused on differentiation toward olfactory placode-like progenitors rather than the formation of fully developed organoids (Bricker et al., 2022). As a result, only a small number of reports have described olfactory organoid-like structures derived from pluripotent cells, and these primarily model early developmental stages rather than mature tissue organization (Frey, 2024).

Whether the neuronal and progenitor-like populations generated in these culture systems faithfully recapitulate the canonical GBC-to-OSN differentiation axis, or instead reflect alternative cellular trajectories that emerge under the selective pressures of *in vitro* culture, remains an important open question. The available evidence suggests that current 3D OE cultures mirror key biological features of the native olfactory epithelium, but they do not yet fully reproduce the complete *in vivo* neurogenic program. The strongest evidence supporting their biological relevance comes from the convergence between marker progression observed in organoid systems and the independently established *in vivo* hierarchy of GBC commitment states (Figure 2), in which sequential expression of Ascl1, Neurog1, and NeuroD1 marks the transition from transit-amplifying progenitors toward neuronal fate (Chen et al., 2004; Durante et al., 2020; Gameiro et al., 2025a). This supports the view that organoid systems capture at least some features of the endogenous commitment program. However, these systems achieve this under experimental conditions that only partially reproduce the native tissue environment: lineage outputs are strongly influenced by media composition, Notch/Wnt/TGF-*β* pathway perturbation, and the absence of the olfactory bulb, lamina propria, and spatial patterning cues that normally govern regional neuronal identity *in vivo* (Kang et al., 2025; Ren et al., 2021). Most studies infer lineage fidelity from marker co-expression at fixed time points rather than from lineage-traced or trajectory-resolved data, and the strongest validation of GBC neurogenic potential continues to come from transplantation and *in vivo* lineage studies rather than from culture systems alone (Coleman et al., 2019). Considerable inter-study heterogeneity has been reported in the identity of isolated cell populations, with mesenchymal and fibroblast-like cells frequently co-isolated alongside neuroepithelial progenitors (Pipolo et al., 2026). Importantly, the alternative and non-neuronal cellular outputs frequently observed in culture should not be dismissed as mere noise or contamination. *In vivo*, GBCs are known to generate non-neuronal lineages following injury, canonical Notch signaling directs neuronal versus non-neuronal fate choice, and disruption of Polycomb repressive complexes can redirect basal cells toward Bowman’s gland identity (Herrick et al., 2018; Ko et al., 2023; Lin et al., 2017). These alternative outputs in culture may therefore reflect genuine latent plasticity. The critical question is therefore not whether cultures generate mixed or alternative cell states, but whether the experimental conditions were designed to model homeostatic neurogenesis, injury-induced regeneration, or progenitor expansion, biological contexts that are rarely distinguished explicitly in the literature. Current evidence therefore suggests that these models are best viewed as biologically informative models of olfactory epithelial niche, particularly for studying progenitor-state regulation and regenerative competence under defined conditions. Nevertheless, faithfully reconstructing the complete *in vivo* GBC-to-OSN differentiation trajectory and mature sensory neuron identity remains a major objective for future organoid and assembloid development.

Overall, while 3D olfactory organoid systems represent an important conceptual and technical advance over earlier culture models, the field remains at an early stage of development. Current systems are not fully consistent, and only partially reflect the structural, functional, and regenerative properties of the native olfactory epithelium. Continued methodological refinement, together with integration of additional cellular and microenvironmental components, will be required to establish olfactory organoids as robust and widely applicable platforms for studying adult neurogenesis, disease mechanisms, and therapeutic interventions.

## Future perspectives: patient-specific “nose-to-brain” models

Despite the significant progress achieved in olfactory epithelial culture systems over recent years, current models still only partially reproduce the complexity of the native olfactory neuroepithelium. Accordingly, the priority for the field is to refine the epithelial organoid itself by improving protocol standardization, preserving the native stem-cell niche, increasing the generation of mature OSNs, and reducing inter-model variability. These objectives are likely to be achieved sequentially, with improvements in epithelial organoid quality enabling functional validation, multicellular integration, and ultimately patient-specific applications.

The first challenge is to improve the epithelial organoid itself by preserving the native stem-cell niche while increasing reproducibility and neuronal maturation. A persistent limitation of current systems is the inability to maintain HBCs in their native quiescent, niche-supporting state. Most existing cultures drive HBCs toward spontaneous activation or lose them through the selective expansion of the more proliferative GBC population during passaging. Recent work in mouse 3D organoid systems has demonstrated that quiescent HBCs can be preserved as a functional niche population actively supporting olfactory neurogenesis rather than being passively depleted, providing an initial methodological template that may be transferred to human biopsy-derived cultures (Gameiro et al., 2025a). In parallel, methodological variation in basal-cell isolation, matrix composition, and growth factor formulation remain the largest source of cross-laboratory variability in organoid outputs. The recent publication of detailed protocol papers for olfactory epithelium organoids supporting neuronal differentiation (Gameiro et al., 2025b) represents an important step toward community standardization, analogous to what the cerebral organoid field achieved with early landmark protocols. Finally, increasing the production of mature OSN-like cells remains a major objective. At present, only a small fraction of cells expresses canonical maturation markers such as OMP and Golf, and robust odorant-evoked responses remain uncommon. Directed differentiation strategies combining timed morphogen exposure with air–liquid interface maturation therefore represent one promising route toward more physiologically mature organoids (Luo et al., 2026; Suzuki et al., 2025).

A second objective will be to demonstrate that these organoids achieve functional maturation rather than simply molecular identity. Electrophysiology, odor-evoked calcium imaging, multi-omics profiling, and integrated neuronal recording platforms should generate standardized functional readouts and enable quantitative comparisons across laboratories. However, even when mature OSN-like cells are produced, functional validation remains technically challenging. An important factor likely limiting the maturation of current organoids is the absence of long-range neuronal connectivity. *In vivo*, OSNs continuously project axons through the cribriform plate toward the olfactory bulb (OB), where synaptic interactions with mitral and tufted neurons contribute to neuronal survival, activity-dependent refinement, and circuit stabilization. The absence of these long-range interactions in isolated olfactory epithelial cultures may limit the acquisition of fully mature neuronal identities and functional sensory properties, motivating the next stage of model development: multicellular and multi-tissue assembloid systems.

Assembloid systems have emerged as a major strategy for modeling higher-order neuronal interactions and circuit-level biology *in vitro* (Sloan et al., 2018). Unlike classical organoids, which largely represent isolated tissue compartments, assembloids combine multiple regional identities and cellular populations into interconnected systems capable of reproducing aspects of neural circuitry, activity-dependent signaling, and intercellular communication. Recent studies in the brain field have demonstrated that assembloids can reproduce functional interactions between distinct neuronal regions while incorporating additional cellular components such as microglia, vascular-like compartments, and immune-associated cells (Wu et al., 2026; Yang et al., 2026). Importantly, these systems reveal biological phenomena that cannot be observed in isolated organoids, including circuit-specific disease phenotypes. The application of these concepts to the olfactory field represents a particularly attractive and still largely unexplored direction. Olfactory assembloids integrating olfactory epithelial organoids with OB–like telencephalic structures may provide a more physiologically relevant environment for OSN maturation by recreating target-derived trophic support, axonal guidance cues, and synaptic connectivity. Such systems could allow the reconstruction of peripheral-to-central sensory circuits *in vitro*, enabling the study of odor processing, axonal targeting, neuronal synchronization, and activity-dependent plasticity within a human context. It should be emphasized, however, that no olfactory-specific assembloid system has yet been reported; feasibility is currently inferred only by analogy to non-olfactory brain assembloid platforms. Whether olfactory epithelial organoids can be functionally integrated into multi-tissue assembloid systems in the manner achieved for cortical or subcortical counterparts remains an open and untested question. This direction is therefore presented here as a conceptually motivated future goal rather than a description of existing systems, offered as a framework to orient the next phase of the field.

To enable these increasingly complex patient-derived models, another major challenge will be the reliable isolation and enrichment of pure olfactory neural progenitor populations. Although minimally invasive nasal swabs and brushing approaches have dramatically increased accessibility, these methods often yield mixtures of respiratory and olfactory epithelial cells. Improved strategies for the selective isolation, expansion, and maintenance of HBCs, GBCs, and other neurogenic populations will therefore be essential for increasing reproducibility and neurogenic efficiency across laboratories. The integration of single-cell transcriptomics, spatial profiling, and surface-marker–guided purification approaches will likely play a central role in refining culture systems. While direct bulk proteomic profiling of raw nasal swabs faces significant challenges due to the overwhelming abundance of extracellular mucus proteins that mask lower-abundance intracellular signals, the incipient field of single-cell proteomics offers a powerful resolution (Sinn and Demichev, 2025). By integrating surface-marker-guided cell sorting or microfluidic isolation, individual neuroepithelial progenitors or sensory neurons can be completely separated from the mucosal matrix prior to mass spectrometry analysis. This allows the field to transition from transcript-level proxies directly to functional protein validation, capturing real-time alterations in protein translation and enzymatic pathways without mucosal interference.

**Figure 3:**
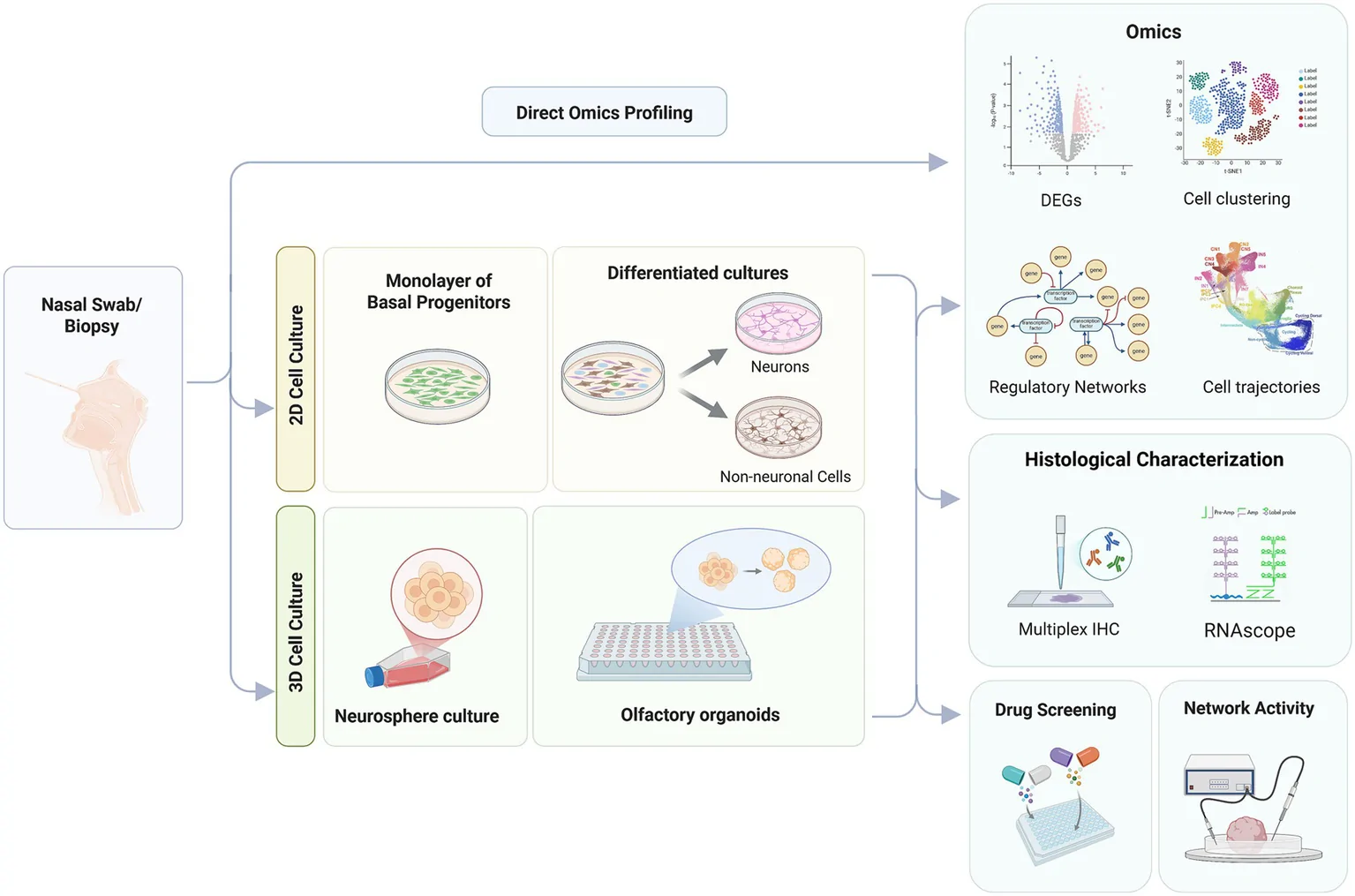
Overview of patient-derived *in vitro* olfactory models and their applications. Human olfactory neuroepithelium obtained through minimally invasive nasal swabs or localized biopsies provides direct access to primary donor-derived tissue for disease modeling, multi-omics profiling, and functional studies. As illustrated in the central workflow, donor material can be directly subjected to molecular profiling approaches, including single-cell transcriptomic and proteomic analyses. Alternatively, patient-derived in vitro models can be established. These include 2D monolayer cultures of basal progenitor cells, which can be differentiated into neuronal and glial cell populations, as well as 3D culture systems such as neurospheres and extracellular matrix-embedded olfactory organoids. As shown on the right, these cultures and their derivatives enable multiple downstream applications, including spatial and molecular characterization by multiplex immunostaining and RNAscope, omics-based analyses that can reveal cellular heterogeneity, differentially expressed genes (DEGs), regulatory networks, and cellular trajectories, personalized drug screening, and functional assessment of neuronal network activity using multi-electrode arrays (MEAs). Created in https://BioRender.com.

The greatest strength of olfactory-derived models may not only lie in their structural complexity, but also in their capacity to preserve patient-specific biological identity. Unlike pluripotent stem cell-based systems, which require extensive cellular reprogramming toward embryonic-like states, olfactory cultures can be established directly from adult patient tissue while maintaining important aspects of the donor’s endogenous molecular and cellular landscape. Although iPSC-derived organoids have transformed the study of human neurodevelopment, the reprogramming process resets epigenetic and transcriptional states, potentially erasing biologically relevant features associated with aging, environmental exposure, inflammation, and disease progression. In addition, iPSC-derived systems primarily model early developmental stages and may incompletely preserve the physiology of mature or regenerating adult neural tissue. In contrast, olfactory-derived cultures originate from an endogenous adult neurogenic niche that undergoes continuous neuronal turnover throughout life. These systems retain aspects of the donor’s genetic background, epigenetic landscape, transcriptional programs, and regenerative state within a living adult neuroepithelial context. This unique property makes olfactory models particularly attractive for studying patient-specific disease mechanisms, longitudinal molecular changes, and personalized therapeutic responses in neuroscience (Figure 3).

As methodologies continue to improve, patient-derived olfactory cultures may become powerful tools for modeling disease-associated cellular phenotypes, monitoring longitudinal molecular changes, predicting therapeutic responses, and developing individualized treatment strategies. Together, the priorities outlined above define a practical roadmap for the next generation of olfactory *in vitro* models, combining HBC-niche reconstitution, protocol standardization, mature-OSN yield optimization, compositional diversification, engineered functional readouts, and olfactory-identity enrichment from minimally invasive samples to address the structural and compositional gaps that currently limit the field. Combined with emerging assembloid technologies and increasingly refined progenitor isolation methods, olfactory platforms may evolve from relatively simple epithelial cultures into integrated patient-specific neural circuit models capable of bridging peripheral sensory biology with central nervous system dysfunction. Ultimately, human olfactory *in vitro* systems bridge the gap between peripheral sensory biology and central nervous system pathology, offering an invaluable, non-reprogrammed foundation for the future of precision medicine.
